# Editorial: Novel Psychoactive Drugs

**DOI:** 10.3389/fpsyt.2019.00119

**Published:** 2019-03-11

**Authors:** Liana Fattore, Aviv M. Weinstein

**Affiliations:** ^1^Institute of Neuroscience-Cagliari, National Research Council of Italy, Cagliari, Italy; ^2^Department of Behavioral Science, Ariel University, Ariel, Israel

**Keywords:** psychoactive drugs, drug abuse and addiction, intoxications, internet drug, legal highs

In the last decade, the trend of drug consumption has completely changed, and an incredibly high number of new psychoactive substances (NPS) have flooded the drug market as legal alternatives to common drugs of abuse. The advent of NPS has contributed to the appearance and growth of a new “drug scenario” characterized by an increased number of intoxicated people presenting with emergencies after consumption of drugs with unknown effects or safety profiles. Indeed, the acute effects of NPS and their long-term side effects are not always known, and safety data regarding their toxicity are often unavailable. Considering that a total of 803 NPS were reported in the period 2009–2017, it is clear that such a situation poses additional challenges for identification, control, and treatment strategies.

We felt that the time has come to deepen and expand our understanding of the peripheral and central actions of NPS and their health consequences. Working as preclinical (LF) and clinical researchers (AW), we have decided to collect leading groups of scientists working in the field to “make the point” of NPS at several levels, from epidemiology to marketing, from clinical to mechanistic studies, including *in vitro* and *in vivo* studies on common (synthetic cannabinoids and cathinones) and less common (2,4-DNP and “hippy crack”) psychoactive substances. As a result, the present Research Topic brings together 16 papers (9 original articles and 7 reviews) of excellent quality and broad impact which cover the main classes of NPS and explore further their toxicology and pharmacology, with a particular emphasis given to the role of NPS in modulating brain neurotransmission and behavior.

According to the last World Drug Report 2018 (United Nations publication, Sales No. E.18.XI.9), by the end of 2017 synthetic cannabinoids and synthetic cathinones represented the largest class of NPS, with 251 and 148 compounds detected, respectively. This Research Topic includes 2 original articles on synthetic cannabinoids, 2 original articles on synthetic cathinones, and 2 reviews summarizing the acute and chronic effects of these two classes of NPS. Specifically, Elmore and Baumann from the Designer Drug Research Unit of NIDA-NIH (Baltimore, USA) used the popular synthetic cannabinoid JWH-018 to expand the current knowledge on the relationship between repeated use of synthetic cannabinoids and serotonergic dysregulation. Their study shows that repeated treatment with JWH-018 induces tolerance to its hypothermic and cataleptic effects and produces transient increases in serotonin 5-HT_1A_ receptor responsiveness without affecting 5-HT_2A_ receptor responsiveness in male rats.

To provide further insights on the mechanisms of action underlying the stimulant effects of synthetic cannabinoids, a multidisciplinary Italian study coordinated by Matteo Marti from the University of Ferrara (Italy), showed that the naphthoylindole compound JWH-018 and the unrelated adamantylindazole AKB48 induced psychostimulant effects in male mice through mechanisms mediated by both cannabinoid CB_1_ and dopamine receptors and likely facilitated the release of ventral striatal dopamine without affecting the activity of the dopamine transporter (DAT) (Ossato et al.).

To update the scenario on synthetic cathinones, Tomáš Páleníček and his productive lab located in Prague (Czech Republic) have contributed to this Research Topic with 4 different contributions. The first provides a detailed investigation of pharmacokinetics and bio-distribution of mephedrone (4-methylmethcathinone, MEPH) and its primary metabolite nor-mephedrone (nor-MEPH) to four different substrates, i.e., serum, brain, lungs, and liver (Šíchová et al.). Authors also demonstrated that methylone (3,4-methylenedioxy-N-methylcathinone) and its primary metabolite, nor-methylone, induced alterations in behavior and body temperature changes that are comparable to MDMA (Štefková et al.). Finding that hyperthermic reaction is more pronounced in the group-housed condition relative to individually housed rats confirms the risk for users to suffer from serotonin syndrome especially when the drug is used in crowded conditions. Moreover, they showed that also 3,4-methylenedioxypyrovalerone (MDPV), a potent pyrovalerone cathinone that recreational users substitute for amphetamines, is rapidly absorbed, readily crosses the blood-brain barrier, is excreted primarily as metabolites and, consistent with its primarily dopaminergic mechanism of action, acts as a typical stimulant with modest hyperthermic and psychomimetic properties (Horsley et al.). Finally, authors provided an exhaustive review on synthetic aminoindanes and discussed their therapeutic potential in medical research along with their pharmacology, behavioral effects, pharmacokinetics, and toxicity (Pinterova et al.).

To summarize the last evidence coming from both animal and human studies on these two main classes of NPS, we provided a comprehensive review on epidemiology, pharmacology, central effects and clinical features, legislation, and regulation of both synthetic cannabinoids and synthetic cathinones. We also discussed why they result in adverse medical and psychiatric effects that seem to be higher than those induced by the natural parent compounds, i.e., cathinone and THC (Weinstein et al.).

But the world of NPS is immense, and other drugs besides cathinones and cannabinoids have been studied. Among them are 3,4-dichloromethylphenidate (3,4-CTMP) and ethylphenidate, two drugs closely related to the attention deficit medication methylphenidate (Ritalin®) that have been reported on online user fora to induce effects similar to cocaine. These two NPS were identified from samples obtained from London dance club amnesty bins or samples purchased on the internet and analyzed by Davidson et al. that here described their neurochemical profile in comparison with their parent compound methylphenidate. The authors showed that while 3,4-CTMP increases the release of dopamine more potently than methylphenidate (likely by blocking the DAT), ethylphenidate possesses a weaker effect and increases dopamine release only modestly while both NPS, like methylphenidate, increase noradrenaline efflux.

Over the last few years, MDMA and its phenethylamine derivatives, such as 2,5-dimethoxy-4-bromo-amphetamine hydrobromide (DOB) and para-methoxyamphetamine (PMA), are also increasingly sold through the internet despite their significant toxicity and widely used as substitutes in “ecstasy” tablets. A collaborative study conducted in Milan (Italy) demonstrated that the oxytocin/vasopressin system modulates the rewarding, prosocial, and anxiolytic effects of MDMA, DOB, and PMA in zebra fish, thus providing an important target for the development of new pharmacotherapies for the treatment of affective disorders caused by MDMA and its phenethylamine derivatives (Ponzoni et al.).

Intriguingly, under the umbrella of NPS, recreational users can find not only newly synthetized substances, but also “old” compounds sold as supplements and weight-loss drugs or typically used in clinical setting, two of which have been investigated by *Andrea Petróczi* and her collaborators of the Kingston University (UK). 2,4-dinitrophenol (2,4-DNP), for example, is an effective but highly dangerous fat burner used in the past for obesity treatment but then withdrawn from the market due to an unacceptably high rate of significant adverse effects. Today, DNP has re-emerged within the body-building community and extreme dieters, particularly among young adults, and is sold mostly over the internet under a number of different names as a slimming aid. Using a sequential mixed method design and based on a hypothetical scenario as if 2,4-DNP was a licensed pharmaceutical drug, the authors elegantly discussed the factors men and women may consider before buying a weight-loss drug such as 2,4-DNP (Bleasdale et al.). Another example of “atypical” NPS is nitrous oxide gas, also known as “hippy crack”' or “laughing gas,” which is a safe, effective, and inexpensive anesthetic used for the management of labor pain, e.g., to decrease fear and anxiety associated with dental procedures or during childbirth. Yet, it is increasingly consumed recreationally for its euphoric, relaxing, and hallucinogenic effects. Authors here reported for the first time trends, awareness, and perceptions of the use of hippy crack among young adults in England and highlighted a worrying willingness of most respondents to use it in the future coupled with a clear lack of awareness of the serious side effects (e.g., psychosis, myeloneuropathy) this gas may cause (Ehirim et al.).

Considering that some medications (e.g., nitrous oxide) are increasingly abused and that some NPS have been tested clinically (e.g., ketamine), it is not fully unexpected that some NPS, based on their known or predicted pharmacology, might have the potential to be clinically useful in brain disorders. To take stock of the situation, in a collaborative (Malaysia, Germany, Switzerland) review, Hassan et al. revisited the existing literature on several NPS for which the neuropharmacological evaluation has made great progress in recent years, including Kratom, Spice, mephedrone and methylone, dimethyltryptamine and novel serotonergic hallucinogens, ketamine and novel dissociative drugs, GHB and GBL, and 1,4-butanediol.

Fentanyl, fentanyl analogs, and other new synthetic opioids with various chemical structures, such as AH-7921, U-47700, and MT-45, have burst relatively recently onto the illegal drug market as NPS. In this Research Topic, Jolanta Zawilska from the Medical University of Lodz (Poland) provided an updated information on the properties of novel synthetic opioids, with a special emphasis given to their acute toxic effects, and reviewed case reports of fatalities involving these drugs.

With two different contributions, Orsolini et al. and Orsolini et al. have discussed the use of NPS in the context of the psychopathology and psychopharmacology of the hallucinogen-persisting perception disorder and reviewed the most commonly abused “psychedelic animals” by combining a search of both scientific literature and online psychonauts' experiences.

To conclude the Research Topic, the French group of *Laurent Karila* and colleagues presented an overview of new technologies in the field of addiction and discussed how they can improve assessment of and interventions in addictive disorders (Ferreri et al.).

The field of NPS is too broad to present a comprehensive overview, but the series of papers in this special issue provide an excellent illustration of the relevant topics, which warranted their publication together. They illustrate the rapidly expanding research on NPS encompassing epidemiological analyses, pharmacological mechanisms, and toxic effects. In light of the great interest that this Research Topic has already attracted and of the increasing number of NPS currently on the market, we have decided to edit in 2019 a follow up Frontiers Research Topic on NPS with the ultimate goal of stimulating research and education in the areas of prevention and treatment. Contributions in any aspect related to the complex world of NPS (e.g., intoxication cases, clinical and animal studies, epidemiology analysis, legislative/regulatory considerations) are welcome.

## Author Contributions

LF and AW contributed equally to this Editorial of the Research Topic on NPS that they edited in 2018.

### Conflict of Interest Statement

The authors declare that the research was conducted in the absence of any commercial or financial relationships that could be construed as a potential conflict of interest.

